# Characterisation of the triple negative breast cancer phenotype associated with the development of central nervous system metastases

**DOI:** 10.3332/ecancer.2016.632

**Published:** 2016-04-11

**Authors:** Katerin Rojas Laimito, Angelo Gámez-Pozo, Juan Sepúlveda, Luis Manso, Rocío López-Vacas, Tomás Pascual, Juan A Fresno Vara, Eva Ciruelos

**Affiliations:** 1 Department of Medical Oncology, Hospital 12 de Octubre, Av. de Córdoba, s/n, 28041, Spain; 2 I+D Department, Biomedica Molecular Medicine, CLAID building, Campus de Cantoblanco, Calle Faraday, 7, Madrid 28049, Spain; 3 Molecular Oncology and Pathology Lab, INGEMM, Hospital La Paz, Spain

**Keywords:** breast cancer, central nervous system, metastases, triple negative

## Abstract

**Aims:**

Breast cancer (BC) is the most frequent tumour in women, representing 20–30% of all malignancies, and continues to be the leading cause of cancer deaths among European women. Triple-negative (TN) BC biological aggressiveness is associated with a higher dissemination rate, with central nervous system (CNS) metastases common. This study aims to elucidate the association between gene expression profiles of PTGS2, HBEGF and ST6GALNAC5 and the development of CNS metastases in TNBC.

**Methods:**

This is a case-controlled retrospective study comparing patients (pts) with CNS metastases versus patients without them after adjuvant treatment. The selection of the samples was performed including 30 samples in both case and control groups. Formalin-fixed, paraffin-embedded samples were retrieved from the Hospital 12 de Octubre Biobank. Five 10 µm sections from each FFPE sample were deparaffinised with xylene and washed with ethanol, and the RNA was then extracted with the RecoverAll Kit (Ambion). Gene expression was assessed using TaqMan assays.

**Results:**

A total of 53 patients were included in the study. The average age was 55 years (range 25–85). About 47 patients (88.67%) had ductal histology and presented high grade (III) tumours (40 patients; 75.47%). Eight women in the case group presented first distant recurrence in the CNS (34.80%), local recurrence (three patients, 13.04%), lungs (two patients; 8.7%), bone (one patient; 4.34%) and other locations (seven patients; 30.38%). In the control group, first distant recurrence occurred locally (six patients; 46.1%), in bone (two patients; 15.4%), lungs (one patient; 7.7%) and other sites (four patients; 23.1%). RNA was successfully obtained from 53 out of 60 samples. PTGS2, HBEGF, and ST6GALNAC5 expression values were not related to metastasis location.

**Conclusion:**

TN tumours frequently metastasise to the visceral organs, particularly lungs and brain, and are less common in bone. The literature suggests that expression of the three genes of interest (PTGS2, HBEGF, and ST6GALNAC5) could be different in TNBC patients with CNS metastasis when compared to patients without it. We did not find a differential expression pattern in PTGS2, HBEGF, and ST6GALNAC5 genes in primary TNBC showing CNS metastases. Further studies are needed to clarify the role of these genes in CNS metastases in TNBC patients.

## Introduction

The incidence of breast cancer (BC) is increasing [1] and has now displaced cardiovascular pathology as the leading cause of mortality among women in the Western world. In Spain, its incidence, mortality and five-year prevalence are 29%, 15%, and 41%, respectively (Globocan 2012), and it is the third most deadly neoplasm in Spain [2, 3] with an annual incidence of 61 cases for every 100,000 women. The triple negative (TN) BC subtype represents approximately 10–20% of all cases of BC in Caucasian women. It characteristically affects young women, is associated with poor prognosis pathological characteristics, high rates of early tumoural relapse, high rates of visceral metastasis (20–30%, particularly lung and brain), short survival and the absence of targeted biological therapy. Additionally, it is related to the basal-like group obtained via genetic analysis and to tumours associated with changes in the BRCA1 gene [12–13, 36]. Tumour genetic changes define the conduct of this tumour and could be responsible for the poor prognosis in this type of patient [7]. Recent studies indicate that shorter survival in the group of patients with BC is due to two characteristics: triple-negative (TN) phenotype and central nervous system (CNS) metastasis [6, 19]. A recent study suggests that the differential expression of some genes is related to the appearance of cerebral metastasis [7].

Genetic expression studies based on studies of levels of mRNA such as the PAM50 (RT-PCR) assay [11] have identified at least four molecular subtypes of BC with different clinical behaviour: Luminal A, luminal B, HER2 and basal-like [4]. Correlating this mRNA study-based classification with an immunohistochemical-based classification shows that most tumours that belong to the basal-like subtype lack expression of oestrogen receivers (ER), progesterone receivers and HER-2 and hence are called triple-negative (TN) tumours [5]. Even though tumours that share an aggressive course are grouped together in the TN group, it is not entirely homogenous; there are at least six subgroups of tumours with different gene expression patterns [12].

The only study (BOS and Col.) that has evaluated additional markers related to the development of metastasis at the cerebral level finds that RE-negative BC cells express three genes (PTGS2, HBEGF, and ST6GALNAC5) in an altered form: these cells have a predisposition to spread at the CNS level. In normal physiological conditions, expression of ST6GALNAC5 is only at the cerebral level [38, 39].

In the present work, we will analyse the expression of three genes of interest (PTGS2, HBEGF, and ST6GALNAC5) in the FFPE tissue of primary TN phenotype breast tumours with CNS metastasis compared to patients who do not present with cerebral metastasis.

## Materials and methods

### Sample design and selection

The study is an analytical observational case (30 samples with CNS metastasis) – control (30 samples without CNS metastasis) study, chosen for similar characteristics in each case. The present study was evaluated and approved by the Hospital 12 de Octubre of Madrid’s institutional ethics committee.

### Patient selection

Patients diagnosed with BC between January 1, 1994 and December 31, 2012 with clinical information and updated clinical follow-up available in the Hospital 12 of October’s archive and documentation service.

### Sample collection

The selection of the biological samples was taken from the Hospital 12 of October Pathological Anatomy Department’s paraffinised tissue bank.

Cases with the TN phenotype with adequate follow-up and with a formalin-fixed and paraffin-embedded (FFPE) tumour sample with at least 50% tumour cells were selected from the pathological anatomy archive. Subsequently, 30 cases that subsequently metastasised to the CNS and 30 cases that did not were taken.

## Sample analysis

### Sample processing

About 10 micron sections of the FFPE tissue blocks were made using a microtome. The RNA was isolated using the Life Technologies RecoverAll Kit following the manufacturer’s protocol. Therefore, the nucleic acids isolated were quantified using UV spectrophotometry.

### Gene expression analysis using RT-qPCR

Quantitative PCR was used to analyse the expression of three genes of interest (PTGS2, HBEGF, and ST6GALNAC5) and two reference genes (IPO8 and POLR2A) validated for use as reference genes for breast cancer using paraffinised tissue [45] using TaqMan assays (ThermoFisher Scientific).

Drawing from the 50–100 ng of RNA extracted from the FFPE samples, a total reverse transcription of 1 ug of RNA was carried out with the Life Technologies High Capacity cDNA Reverse Transcription Kit following the aforementioned company’s TaqManGene Expression Assays Protocol.

**Table:** 

Gene	Assay ID
IPO8	Hs00183533_m1
POLR2A	Hs00172187_m1
PTGS2	Hs00153133_m1
HBEGF	Hs00181813_m1
ST6GALNAC5	Hs00229612_m1

### Statistical analysis

These expression data (Ct values) were obtained in triplicate for each sample, and the mean *C*t was calculated. The missing values were replaced with a maximum *C*t value set at 40. Subsequently, the expression values of the genes of interest were standardised using the Δ*C*t method, which consists of calculating relative expression values as differences between a normalisation factor, which in this case is the geometric mean of the expression of both reference genes, and the mean CT value of each gene. Later, a constant was added and a relative expression value for each gene in each sample was obtained, in which an increase in one supposes double the expression. In order to evaluate whether there is a differential expression of the genes of interest in TN breast cancer patients with CNS metastasis vis-à-vis the group without CNS metastasis, the Mann–Whitney U-test was applied.

## Results

### Clinical characteristics of the patients

The median age of the patients in the study was 55 years (range 25–85). In this group of patients, 47 (88.67%) were of the ductal histologic type and 40 patients (75.47%) were histologic Grade III. Tumour size and nodal involvement are summarised in Table 1.

In the case group, eight patients (34.80%) had cerebral metastasis as the first site of recurrence, three patients (13.04%) had local recurrence, two patients (8.7%) lung, one patient (4.34%) bone, and seven patients (30.08%) other locations. In the control group, six patients (46.1%) had local relapse as the first site of remote recurrence, two patients (15.4%) bone, one patient (7.7%) lung, and four patients (23.1%) other sites.

### Evaluation of gene expression (PTGS2, HBEGF, ST6GALNAC5)

**Figure 1.**
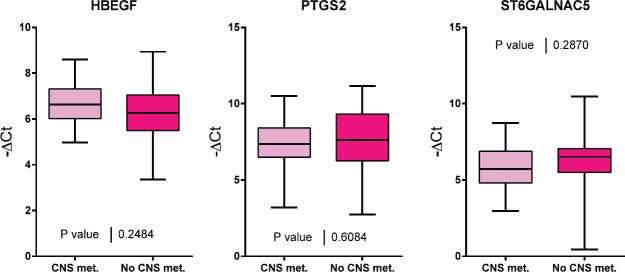
A comparison of HBEGF, PTGS2 and ST6GALNAC5 gene expression in tumour samples from patients with and without CNS metastasis.

Of the 60 selected samples, a sufficient amount of RNA was extracted to carry out experiments on 53 samples. PTGS2, HBEGF, and ST6GALNAC5 gene expression was evaluated for the two groups of interest (Figure 1). No significant differences were found in the expression of these three genes between both groups of patients.

## Discussion

BC, as previously mentioned, is a genetically heterogeneous disease and its clinical incidence, characteristics, and prognosis differ significantly by ethnicity and race [21]. Studies carried out in North America find that Latina patients have a lower incidence but also greater mortality from BC than Caucasian patients. Also, the rate of TN tumours is significantly greater in this group of patients [22–34].

The metastasis process is complex and includes cellular intravasation, survival in the circulation, extravasation to a distant organ, angiogenesis and uninhibited growth in the host tissue [8]. Tumour gene expression studies find that the expression of some genes enables tumour cells and predisposes them to dissemination in a specific form to organs like the lungs [9–10].

Exploration of this pathology has begun to produce results and a variety of studies have found responses, albeit modest ones, to drugs like anti-EGFR (cetuximab and erlotinib), SRC inhibitors (dasatinib) and anti-angiogenics (bevacizumab) [14]. Additionally, drugs that target the PI3K/PTEN/AKT pathway and Notch survivin are presently being studied [14, 16, 17].

The epidemiological evaluation made by our group of the patients with BC seen at the 12 de Octubre University Hospital found that the TN phenotype, in concurrence with the American reports, represents around 20% of breast tumours [22].

The development of cerebral metastasis differs from that of other locations due to particularities like the special composition and density of the cerebral parenchyma and the high impermeability of the blood–brain barrier (BBB) produced by the complexity of the structures that form it, including tight junctions, the absence of fenestrations and very low pinocytic activity, as well as an extracellular matrix, pericytes, and astrocytic foot processes. The cerebral capillaries also have a high electrical resistance that increases the impermeability of this membrane to the polar and ionic substrata. Added to this is a set of extraction transporters that includes *p*-glycoprotein, MRP-1 to 6, breast cancer-resistant protein (BCRP), and organic anion and cation transporters [20]. All this causes systemic therapies to pass through the BBB, and therefore, their access to metastasised parts of the brain is low and insufficient to guarantee the effectiveness of most of the treatments available.

Bos *et al* selected RE-negative BC cells with a high predisposition to infiltrate the brain and evaluated the expression of more than 240 genes in them. The process included the inoculation of these cells in murine models, and the selection of those cells with high capacity to develop cerebral metastasis. After identifying genes through an *in vivo* model, their role was evaluated on the basis of breast tumour data and a group of genes related to the development of cerebral metastasis in RE-negative breast tumours was thus selected. They found that the altered expression of 17 genes was associated with the development of cerebral metastasis. Three of these genes had high expression: cyclooxygenase COX2, an EGFR ligand and the α2, 6-sialyltransferase ST6GALNAC5 gene. The identification of the COX2 gene in this group demonstrates the importance of the inflammation process in the development of brain metastasis, whereas genes related to the epithelial growth factor (EGF) are related to the replication capacity of the tumour cell. Finally, sialyltransferases are related to cell–cell interactions and change in this capacity could be related to the capacity to produce remote metastasis [7].

TN tumours frequently metastasise to the visceral organs, particularly the lungs and brain, and less frequently at the bone level. The literature suggests that the expression of the three genes of interest (PTGS2, HBEGF, and ST6GALNAC5) must be different in patients with TNBC that developed metastasis at the cerebral level when compared to patients who do not develop CNS metastasis.

We did not find a differential expression pattern in these genes (PTGS2, HBEGF, ST6GALNAC5) in the primary breast tumour that developed cerebral metastasis. This could be due to the fact that cerebral metastases host genetic alterations different from the ones observed in the primary tumours; this is still unknown. Priscilla *et al* sequenced the exome of 86 cerebral metastases paired with the primary tumour tissue and normal tissue, finding clinically informative changes in the cerebral metastasis that were not found in the primary tumour tissue sample in 53% of the cases. The genes involved in the development of cerebral metastasis with greater frequency were TP53, PIK3CA, GATA3 and other mutated genes in lesser frequency including AKT1, CDH1, MAP3K1, PTEN, CDH1, RB1, and CDKN1B. They concluded that activation of the PI3K/AKT/mTOR pathway and CDK may be involved in the development of CNS metastasis [40].

The study of patients with TNBC who carry the BRCA-1 and BRCA-2 mutation is one analysis pathway, as these tumours are sensitive to PARP (polyadenosine diphosphate ribose polymerase 1) inhibitors like Olaparib, which penetrates the BBB [15, 44].

Other authors have suggested that androgens, directly activating astrocytes in the cerebral microenvironment, can facilitate the establishment of neoplastic cells originating in TN breast tumours. This would explain why younger TN patients, with greater oestrogen levels, have a greater risk of cerebral metastasis [41]. These findings could justify the absence of differences in gene expression between cases with the presence of cerebral metastasis and those that do not develop it, since the development of these metastases will depend more on the hormonal profile of the patient than on intrinsic factors of the tumour cell.

Other researchers also suggest elements external to the tumour itself as they show that the methylation level is unchanged in TN breast tumour cells in comparison with other BC phenotypes when they metastasise to the brain [42]. Stirzaker *et al* indicate that characterising methylation patterns could help us identify predictive biomarkers in the future [43].

## Conclusion

In conclusion, our findings highlight that not only are the differences in gene expression important when it comes to predicting the risk of cerebral metastasis but also other aspects like oestrogen level could play an important role. Future studies are needed to clarify the role of these genes in primary breast tumours in patients with TNBC who develop cerebral metastases.

## Figures and Tables

**Table 1.** Clinical characteristics of the tumour.

	Patients (pts)	%
***T*_1_**	9	16.98
***T*_2_**	34	64.15
***T*_3_**	6	11.32
***T*_4_**	4	7.54
***N*_O_**	20	37.73
***N*_1_**	13	24.52
***N*_2_**	6	11.32
***N*_3_**	14	26.41
